# Experimental data for the rate of CO_2_ release from seawater under vacuum at 30°C and ambient pressure at 100°C

**DOI:** 10.1016/j.dib.2025.112435

**Published:** 2026-01-03

**Authors:** Paul Straatman, Matteo Gazzani, Wilfried van Sark

**Affiliations:** Utrecht University, Copernicus Institute of Sustainable Development, Princetonlaan 8a, 3584 CB Utrecht, The Netherlands

**Keywords:** Carbon dioxide, Separation, Depressurization, Kinetics, Experimental data

## Abstract

This dataset provides experimental measurements to quantify the rate of CO₂ release from (sea)water. Two different conditions were tested: vacuum pressure and 30°C and ambient pressure and 100°C. Data were collected using 1) a vacuum setup consisting of vacuum flask, placed in a thermostat bath at 30°C connected to a vacuum pump, protected by a cold trap and 2) a beaker without vacuum setup for the atmospheric experiments. Rates were inferred by measuring pH and the total inorganic carbon (TIC) in the water. The latter was measured ex-situ using a TIC analyzer. The TIC concentrations were corrected for reduced volume of the residue to the original volume to determine the actual CO_2_ release after each timestep. Actual seawater samples were utilized to determine the relationship between CO₂ release and water residence time under vacuum and ambient pressure boiling circumstances. The dataset includes variables such as CO₂ release rates and pH changes over time that the sample was subject to boiling conditions, providing valuable insights for designing process equipment for marine Carbon Dioxide Removal (mCDR) applications (for example in evaporative desalination processes) in both atmospheric and sub atmospheric pressures.

Specifications Table

**Table:** 

Subject	Earth & Environmental Sciences
Specific subject area	Marine Carbon Dioxide Removal (mCDR)
Type of data	Experimental measurements of Total Inorganic Carbon and pH.
Data collection	The data were collected using a vacuum flask setup consisting of a thermostat bath [Fisherbrand FB15049], beaker and hot plate for atmospheric boiling, total organic carbon [TOC] analyser Shimadzu TOC-V(CPH) And ASI-V autosampler, and calibrated pH meter SI series [SI400 7400-005]
Data source location	Seawater samples taken at the Dutch shoreline of the city of Hague; measurements carried out at the Geoscience lab of Utrecht University (Utrecht)
Data accessibility	Repository name: Mendeley data Data identification number: Mendeley Data, V1, doi:10.17632/x5vpypjzwp.1 Direct URL to data: Experimental data for the rate of CO₂ release from seawater under vacuum at 30°C and ambient pressure at 100°C - Mendeley Data Instructions for the raw data: please choose “download all” to access the raw data.
Related research article	Straatman, P. J. T., & van Sark, W. G. J. H. M. (2021). Indirect air CO_2_ capture and refinement based on OTEC seawater outgassing. *iScience, 24*(7), 1-13. Article 102754. https://doi.org/10.1016/j.isci.2021.102754

## 1 Value of the Data

• The dataset provides empirical measurements of CO₂ release from seawater under boiling conditions, essential for validating theoretical models of marine Carbon Dioxide Removal (mCDR) processes that work with evaporation of seawater, such as Ocean Thermal Energy Conversion (OTEC), multi effect (seawater) distillation (MED), and mechanical vapour recompression (MVR) desalination.
• Researchers and engineers can utilize these data to design and optimize equipment where CO_2_ release from natural water solutions is relevant, for example for mCDR processes coupled to evaporative desalination.
• The data facilitate a better understanding of the kinetics of CO₂ release during depressurization, contributing to the development of efficient carbon removal technologies.

## 2 Background

The rising atmospheric concentration of carbon dioxide (CO₂) is a major driver of global warming [1]. Despite clear evidence of severe and lasting ecosystem impacts, current trajectories point toward surpassing 1.5°C of warming [2]. To mitigate these effects, various CO₂ removal strategies are under development [3]. One option is capturing CO₂ dissolved in seawater, achieving Indirect Air Capture (IAC) through the ocean, also termed marine Carbon Dioxide Removal (mCDR) [4].

Electrochemical cells have been proposed to separate CO₂ from seawater [5], while vacuum CO₂ degassing in Ocean Thermal Energy Conversion (OTEC) cycles offers an alternative pathway [6]. However, prior theoretical work largely relied on equilibrium models, neglecting dynamic aspects such as reaction and water residence times. Experimental validation of these kinetics is crucial for designing and scaling process equipment effectively.

In particular, the rate of CO₂ release during seawater evaporation under vacuum remains poorly understood. This is relevant for Open Cycle OTEC systems and thermal desalination, where seawater is exposed to vacuum conditions at ambient tropical temperatures (25–30°C). This study therefore aims to experimentally quantify CO₂ release dynamics as a function of residence time, providing essential data for advancing mCDR technologies.

## 3 Data Description

**Fig. 1:**
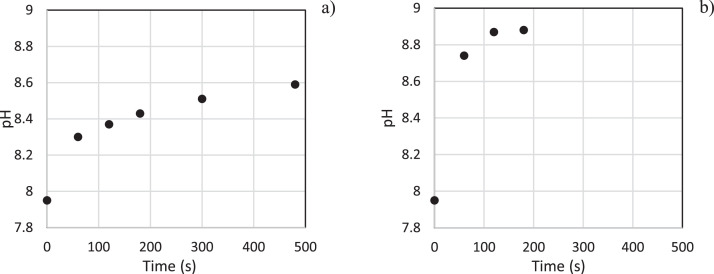
pH increase vs time. (a) vacuum pressure and 30.0°C; at *t* = 0, all air was removed and the vapor pressure equaled the pressure in the flask being 20 mbar absolute ± 5 mbar, as the liquid started to boil from that moment. (b) atmospheric pressure and 100°C. At *t* = 0, the vapor pressure equaled the atmospheric pressure in the beaker, as the liquid started to boil from that moment.

**Fig. 2:**
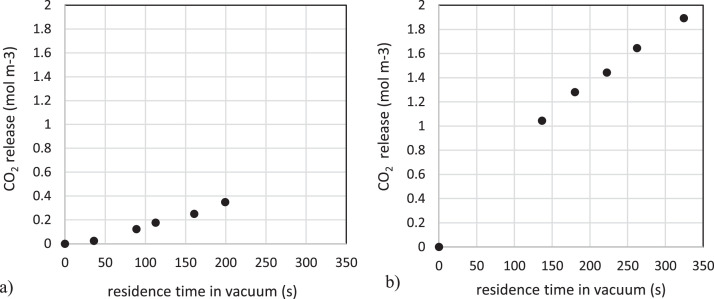
Time dependent CO₂ release from seawater. (a) left, vacuum pressure (20.0 mbar) and 30°C; (b) right, atmospheric pressure and 100°C.

Data are reported in the following order: Tables 1, 2 and Fig. 1 report pH measurements; Tables 3, 4 and Fig. 2 report the total inorganic carbon measurements. Data are listed according to sampling time (see section 4 for methods). Section 4 describes the experiment methods.

**Table 1:** Data of pH measurements as a function of time at 30.0°C and vacuum pressure (doi:10.17632/x5vpypjzwp.1).

Point	t_residence_ (s)	Pressure (mBara)	pH
1	0.00	20.0	7.95
2	60.0	20.0	8.30
3	120	20.0	8.37
4	180	20.0	8.43
5	300	20.0	8.51
6	480	20.0	8.59

**Table 2:** Data of pH measurements as a function of time at 100°C and ambient pressure (doi:10.17632/x5vpypjzwp.1).

Point	t_residence_ (s)	Pressure (mBara)	pH
1	0.00	1010	7.95
2	60.0	1010	8.74
3	120	1010	8.87
4	180	1010	8.88

**Table 3:** Dataset for determination of time dependent CO₂ release under vacuum at 30.0°C with actual seawater. (content of the related field dataset doi:10.17632/x5vpypjzwp.1).

Sample ID	C_t_ (mg L^-1^)	V_t_ (ml)	C_corr_ (mg L^-1^)	n_C_ (mol m^-3^)	t_residence_ (s)
TC-TIC1289	32.3	200	32.3	0.000	0.00
TC-TIC1290	32.3	198	32.1	0.024	36.0
TC-TIC1291	33.2	186	30.9	0.123	88.8
TC-TIC1292	34.1	177	30.2	0.176	113
TC-TIC1293	34.7	169	29.3	0.250	161
TC-TIC1294	37.9	149	28.2	0.349	199

**Table 4:** Dataset for determination of time dependent CO₂ release at 100°C with actual seawater. (content of the related field dataset doi:10.17632/x5vpypjzwp.1).

Sample ID	C_t_ (mg L^-1^)	V_t_(ml)	C_corr_ (mg L^-1^)	n_C_ (mol m^-3^)	t_residence_(s)
TC-TIC1295	32.3	200	32.3	0.00	0.00
TC-TIC1296	31.7	125	19.8	1.05	137
TC-TIC1297	33.9	100	17.0	1.28	180
TC-TIC1298	37.5	80.2	15.1	1.44	222
TC-TIC1299	42.0	59.9	12.6	1.65	262
TC-TIC1300	38.5	49.9	9.61	1.90	324

Table 1 reports the experimental measurement of pH at different time steps under vacuum and at 30.0°C. Table 2 reports the experimental measurement of pH at different time steps at ambient pressure and 100°C. The vapor pressure of seawater at 30 C is around 42 mBara, but the indicator showed ± 5 mBara around 20 mBara.

In Fig. 1(a), the pH of the seawater sample is plotted against time of residence in vacuum at 30.0°C. Notably, the pH adapts to the vacuum, finding a new equilibrium as CO₂ concentration decreases. In the beginning of the experiment, mostly molecular dissolved CO_2_ is rapidly evading the solution following Henry’s law. However as the experiment continues, bicarbonate is converted to molecular CO_2_ in which the conversion reaction of bicarbonate is slower than the degassing of molecular CO_2._ Although the pH stabilizes over time, bicarbonate is consumed to form CO_2_ and carbonate. CO_2_ still evades the solution, so along the experiment the total inorganic carbon is reduced.

Table 3 reports the experimental results of the TIC measurement at 30.0°C. It is observed that the actual concentration of TIC of the residue seawater in the vacuum flask increases with time. However, to calculate the amount of CO₂ that has evaporated from the solution, it is required to calculate the TIC concentration in the situation of the original sample volume, as some of the original volume of water has evaporated during the experiment. Therefore, the residue volume reduction was measured at every timestep by weighing the vacuum flask, with 1 g or 1 ml accuracy. The flask including stops was weighed empty and dry first to know the weight of the flask. The sample pots containing the samples that were taken for TIC were directly closed after taking them.

In Table 4, the experimental results of the TIC measurement at atmospheric pressure and 100°C are presented.

Using the data shown in Fig. 2, it is possible to compute an empirical average CO_2_ release rate over the experiments total time, which corresponds to 1.7 × 10^-3^ mol m^-3^ s^-1^ for 30°C, and 5.8 × 10^-3^ mol m^-3^ s^-1^ for 100°C. The higher value at higher temperature is consistent with thermodynamics. The data presented can be used to estimate the CO_2_ release of seawater undergoing a change in temperature and pressure, for example vacuum desalination processes. Using Fig. 2, a skilled designer can size a flash evaporator vessel, a component of thermal desalination systems, for having sufficient residence time to obtain a desired yield of CO_2_.

## 4 Experimental Design, Materials and Methods

### 4.1 Seawater sample collection

A seawater sample was collected from the North Sea, 52°04′05.6"N 4°12′52.0"E, at a depth of 0.2 meters below the surface off the coast of The Hague, the Netherlands, in October 17 ^th^, 2024. The sample was stored in an airtight, fully filled container to minimize atmospheric gas exchange before the experiment, and stored at 4°C until analysis. No filtration or additional pre-treatment was applied. For the offline TIC measurement, the samples were put in 25ml vials with a Teflon lined cap so as to seal the sample from air before processing. The initial TIC concentration of the sample used in the experiments was 32.3 mg C L⁻¹. While salinity and alkalinity were not measured directly, typical values for this location and season are S = 28–32 PSU and TA = 2330–2520 µmol kg⁻¹, based on regional observational datasets [7].

### 4.2 Experimental setup

**Fig. 3:**
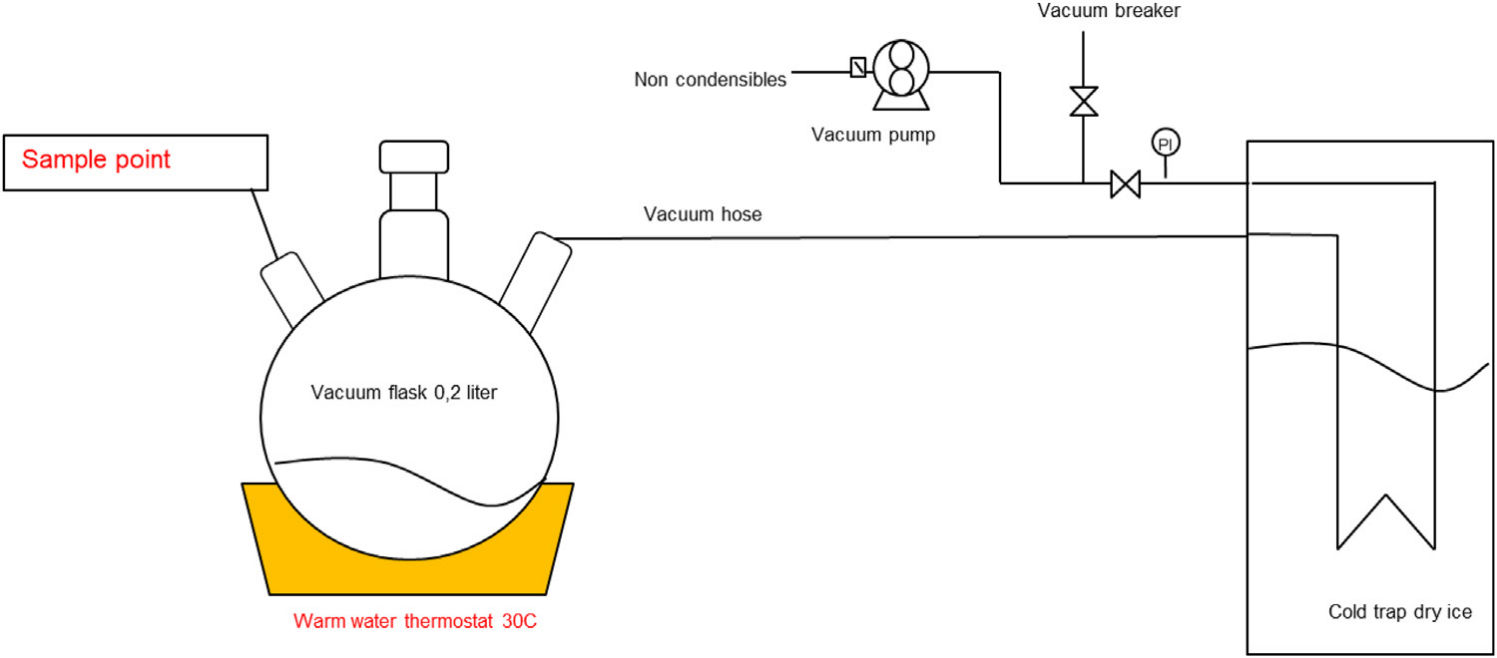
Schematic representation of the vacuum setup.

The main experimental setup (see Fig. 3) consists of a round-bottom 500 mL flask with three ports. The first is a sampling port sealed with a rubber septum, which allows for the extraction of small liquid samples using a syringe while preventing air from getting in contact with the sample. The second port is the vacuum connection. A tube connects the flask to a vacuum pump via a cold trap [KGW isotherm cold trap], filled with dry ice, which condenses any evaporated water before reaching the pump. A pressure gauge is installed along the vacuum line to monitor the system pressure. The third port is sealed port which prevents atmospheric air ingress and ensures controlled pressure conditions. The temperature is kept constant at 30°C using a thermostat bath [Fisherbrand FB15049].

A 200 mL seawater sample was introduced into the flask. The vacuum pump was activated to reduce the pressure, inducing boiling at approximately 30°C, corresponding to the water’s vapor pressure. At regular time intervals (Δt), a small liquid sample (5 mL) was withdrawn via a syringe and the remaining volume was measured by weighing. TIC was measured for each withdrawn sample. TIC was quantified using a Shimadzu TOC/TIC analyzer, where inorganic carbon is released by phosphoric acid addition and detected by infrared absorption. Analytical accuracy was ensured through a multi-point carbonate calibration curve, procedural blanks, duplicate injections, and routine QC samples, following the laboratory’s established operating procedures. TIC values were corrected for the original volume, accounting for the gradual reduction in liquid phase volume due to evaporation. The recorded TIC values were plotted against residence time under vacuum. The observed trends were analyzed to determine the relationship between TIC depletion and water loss over time. For the measurements at 100°C and atmospheric pressure, this setup was simplified, by replacing the flask by a beaker, and a hot plate as heat source, while keeping the sampling method the same. Time was measured using a stopwatch app on the smartphone, with 1 second accuracy.

Calibration procedure: We use blanks, A 4-point standard, with 3-5 duplicates per standard. For every run, 2 – 5 QC samples, with either IC and OC, or only OC depending on the total number of samples.

Detection limit CPN series: 50 microgram per liter.

Reproducibility / accuracy: 1.5 % max.

### 4.3 pH measurement procedure

pH was measured using the same setup for vacuum boiling of seawater as in Fig. 3. A 200 mL seawater sample was introduced into the flask. The vacuum pump was activated to reduce the pressure, inducing boiling at approximately 30°C, corresponding to the water’s vapor pressure. At regular time intervals (Δt), a small liquid sample (5 mL) was withdrawn via a syringe. pH was measured for each withdrawn sample with a calibrated pH sensor: SI series pH meter [SI400 7400-005] (± 0.1 pH), and rinsed with demi water after each measurement. pH values were plotted against time. Before measuring pH of the samples, they were cooled to 25°C. The pH sensor was calibrated using 3 buffer solutions at pH 4.00, 7.00, and 10.0.

### 4.4 TIC calculation procedure

The TIC concentration (± 0.001 mg TIC/l) at time *t C_t_* was corrected by:

(1) $$\begin{array}{c} C_{\text{Corr},t}=\frac{V_{t}}{V_{0}}\times C_{t} \end{array}$$

where *V_t_* is the volume at time *t* (L) and *V*_0_ the starting volume (L). The release of carbon mass per m^3^ seawater is expressed as m CO_2_, is calculated as follows:

(2) $$m_{CO_{2}}=C_{t0}-C_{\text{corr},t}$$

Where C_t0_ is the original carbon concentration in seawater at the start of the experiment, in mg L^-1^. This converts to moles of carbon *n*_C_ as following:

(3) $$n_{C}=\frac{m_{C}}{M_{C}}$$

Where M_C_ is the molar mass of Carbon, 12 g mol^-1^, and since 1mol CO_2_ is 1 mol C, it follows that *n*_C_ = *n*_CO₂_ in mol m^-3^, and *V*_t_ is the volume of the sample at time t, obtained by weighing the flask, preventing air in contact with the sample.

## Limitations

The experimental setup did not account for additional influencing factors such as temperature variations, interface turbulence, or external enhancements to mass transfer, such as ultrasonic agitation or increased heat exchange surfaces. These factors could significantly impact the kinetics of CO₂ release and should be considered in future research.

For industrial applications, where optimizing separation processes is essential, an extensive investigation into the interplay between heat transfer, evaporation dynamics, and reaction kinetics is recommended. Systematic studies incorporating varying thermal inputs, different vacuum conditions, and potential enhancements to mass transfer would provide deeper insights into optimizing CO₂ removal efficiency.

This study aimed to determine the relationship between residence time and CO₂ release during vacuum degassing of seawater. The results indicate that CO₂ release follows a progressive process influenced by both the initial outgassing of dissolved molecular CO₂ and the subsequent equilibrium shifts in the bicarbonate-carbonate system. The experimental observations suggest a link between CO₂ release and water evaporation, but the possible dependence of CO₂ release rate on evaporation rate was outside the scope of this study.

Further research is required to fully optimize the controlling mechanisms and separation processes for mCDR applications.

## Ethics Statement

The authors have read and followed the ethical requirements for publication in Data in Brief and confirmed that the current work does not involve human subjects, animal experiments, or any data collected from social media platforms.

## Credit Author Statement

Paul Straatman: visualization, conceptualization, methodology, performance of sample preparation and drafting of article. Matteo Gazzani: editing the document and supervision and co-interpretation of experiments. Wilfried van Sark: final editing and general supervision.

## Data Availability

Mendeley DataExperimental data for the rate of CO₂ release from seawater under vacuum at 30 °C and ambient pressure at 100 °C (Original data).

## References

[1] Pathak M., Shukla P.R. (2022). Contribution of Working Group III to the Sixth Assessment Report of the Intergovernmental Panel on Climate Change.

[2] Reisinger A., Fuglestvedt J.S., Pirani A., Geden O., Jones C.D., Maharaj S., Poloczanska E.S., Morelli A., Johansen T.G., Adler C., Betts R.A., Seneviratne S.I. (2025). Overshoot: a conceptual review of exceeding and returning to global warming of 1.5°C. Annu. Rev. Environ. Resour..

[3] Prütz R. (2023). Understanding the carbon dioxide removal range in 1.5°C compatible and high overshoot pathways. Environ. Res. Commun..

[4] Doney S. (2025). The science, engineering, and validation of marine carbon dioxide removal and storage. Annu. Rev. Mar. Sci..

[5] Sullivan I., Goryachev A., Digdaya I.A. (2021). Coupling electrochemical CO₂ conversion with CO₂ capture. Nat. Catal..

[6] Straatman P.J.T., Van Sark W.G.J.H.M. (2021). Indirect air capture and refinement based on OTEC seawater outgassing purification of CO₂. iScience.

[7] Norbisrath M., van Beusekom J.E.E., Thomas H. (2024). Alkalinity sources in the Dutch Wadden Sea. Ocean Sci..

